# Non-epileptic Seizures versus Frontal Lobe Epilepsy in an Adolescent: A Case Report

**DOI:** 10.7759/cureus.5732

**Published:** 2019-09-23

**Authors:** Sonia Gaur

**Affiliations:** 1 Psychiatry, Stanford University, Stanford, USA

**Keywords:** topiramate, psychogenic non-epileptic seizures, inpatient hospitalization, nocturnal frontal lobe epilepsy, vasovagal syncope, adolescent psychiatry, conversion disorder, polypharmacy, electroencephalogram, mri

## Abstract

Multiple inpatient psychiatric hospitalizations can be due to system issues, patient complexity, family dynamics, and misdiagnoses to name a few. This study highlights a diagnostically challenging case and how that, in itself, contributed to hospital admissions. Although 18 months elapsed from the time of the initial presentation to the diagnosis of non-epileptic seizures (NES), the suspicion of the diagnosis may have been made earlier by clinicians. The evidence for seizures of post-ictal confusion followed by lethargy, amnesia for the event, and response to an anti-seizure medication only could have provided a higher index of suspicion for NES. Many health care providers will argue that this will create over-diagnoses of NES and usage of anti-epileptic medications. While reviewing the literature on NES, it was noted that frontal lobe epilepsy (FLE) causing psychiatric comorbidities has been poorly studied. Furthermore, this case highlights that within the field of child psychiatry, the same clinical presentation can be interpreted differently. This case helps us understand how eliciting clinical information to enable the timely ordering of imaging could help in diagnoses. This may help set up clinical guidelines for NES for the mental health providers to facilitate improvement in diagnoses and treatment.

## Introduction

Non-epileptic seizures (NES) are paroxysmal events with both objective and subjective findings with minimal to absent correlation of electroencephalogram (EEG) findings with the event [1]. Moreover, they may present with epilepsy and/ or conversion disorder, thereby making it difficult to tease the foci from non-specific findings. Although multiple factors contribute to morbidity, this case highlights the need to be more aware of the complexities of frontal lobe epilepsy (FLE).

## Case presentation

A 16-year-old female has had multiple inpatient psychiatric hospitalizations for danger to self and others over a period of 18 months. She presented to the author in her eighth hospitalization. The presentation was consistently episodic in nature, that is, she initially and partially responded to several antipsychotics and anti-depressants. However, there were breakthrough episodes that increased in intensity and the time in between episodes reduced with time. She showed limited response to valproic acid and oxcarbazepine but responded to topiramate.

With the episodes being ultra-rapid in presentation, usually resulting in involuntary hospitalization for danger to self or others and being aggressive and disturbing to the observer, behaviors were captured over a period of multiple hospitalizations. These episodes followed a pattern, as enlisted below:

• Motor changes: She would contract her muscles, creating a grimacing facial expression (sardonic smile), with the head tilting to one side, followed by a posture that involves an aggressive stance of crouching with growling, guttural sounds. This created a significant fear in every person who initially observed the episode.

• Followed by complex motor functions: she could walk, open and close drawers, and she threw objects already in her hand, scratched walls, and banged her head. 

• Awareness: She claimed to see people talk to her and occasionally was able to follow simple instructions like drinking water, but has no memory of these actions afterward. 

• Grounding technique worked when the episode was triggered in a controlled setting.

• When medicated with topiramate, she would be able to request to go into the quiet room if she felt the episodes were coming.

• She would try to bite others and used an object she had in her hand; on one occasion, it was a knife, resulting in involuntary hospitalization.

• Post episode: Amnesia for the event, with a period of confusion, disoriented, followed by sedation and not able to follow instructions: color the picture. This could last for a few minutes to sometimes an hour.

• Violent and aggressive themes in her nightmares.

• Nocturnal enuresis.

**Figure 1:**
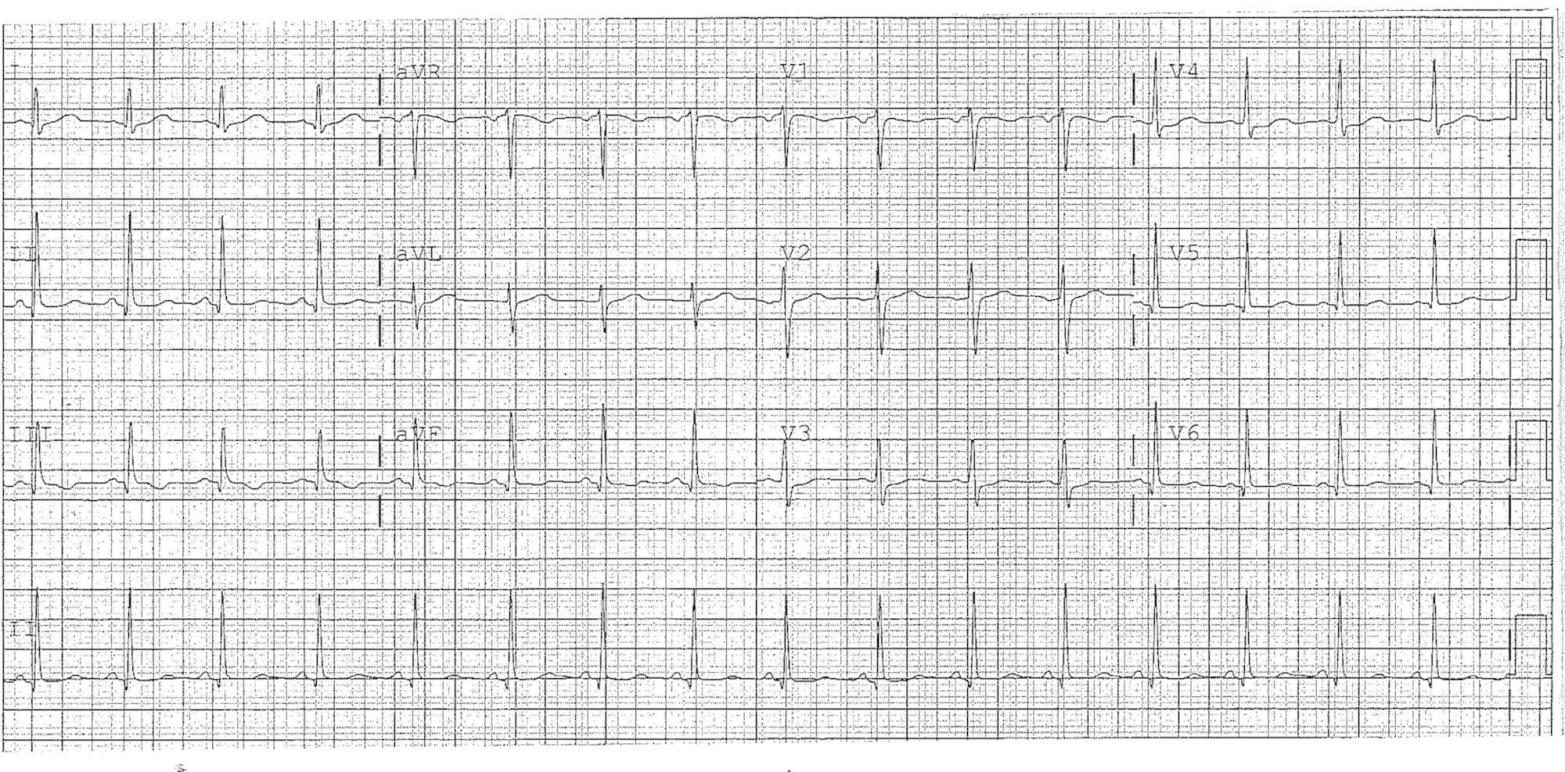
Normal EKG EKG, electricardiogram

Inpatient testing was limited and consisted of Trauma Symptom Checklist for Children, WISC-V, Roberts-2 that revealed a low IQ (89), slow processing speed (<10%) with aggressive and sexual prominent themes. Her early history was significant for being separated from her biological mother when she was one year old, due to neglect. She was more attached to her biological father and stepmother. Her performance at school was below grade level with limited peer interactions. She was likable, quiet, and works well with few adults. Her original and subsequent presentations were of vasovagal attacks and nocturnal enuresis, resulting in urinary tract infections. She was observed by outpatient neurology and cardiology during these episodes without any conclusive diagnoses. Her blood work to rule out any infectious and immune etiologies was unremarkable. The EKG (Figure 1) on different occasions was unremarkable. Her father was diagnosed with Churg Strauss disease, but her eosinophil count was always within normal limits. Her paternal aunt had similar episodes and was stabilized at age 50 years, with three anti-convulsants.

Medication trials of escitalopram 30 mg, fluoxetine 40, aripiprazole 30 mg, chlorpromazine 150 mg, oxcarbazepine 300 mg, valproic acid (101 mcg/ml), olanzapine 30 mg, and Ativan up to 4 mg po/im were ineffective alone or in combinations. Multiple diagnoses of depression, PTSD, dissociative identity disorder, NES, obsessive-compulsive disorder, borderline IQ, depression, psychosis not otherwise specified, conversion, cluster B traits, and cycloid psychosis were considered at different times. She was discharged to the residential facility with the diagnoses of NES and depression on topiramate 350 mg and fluvoxamine 300 mg.

**Figure 2:**
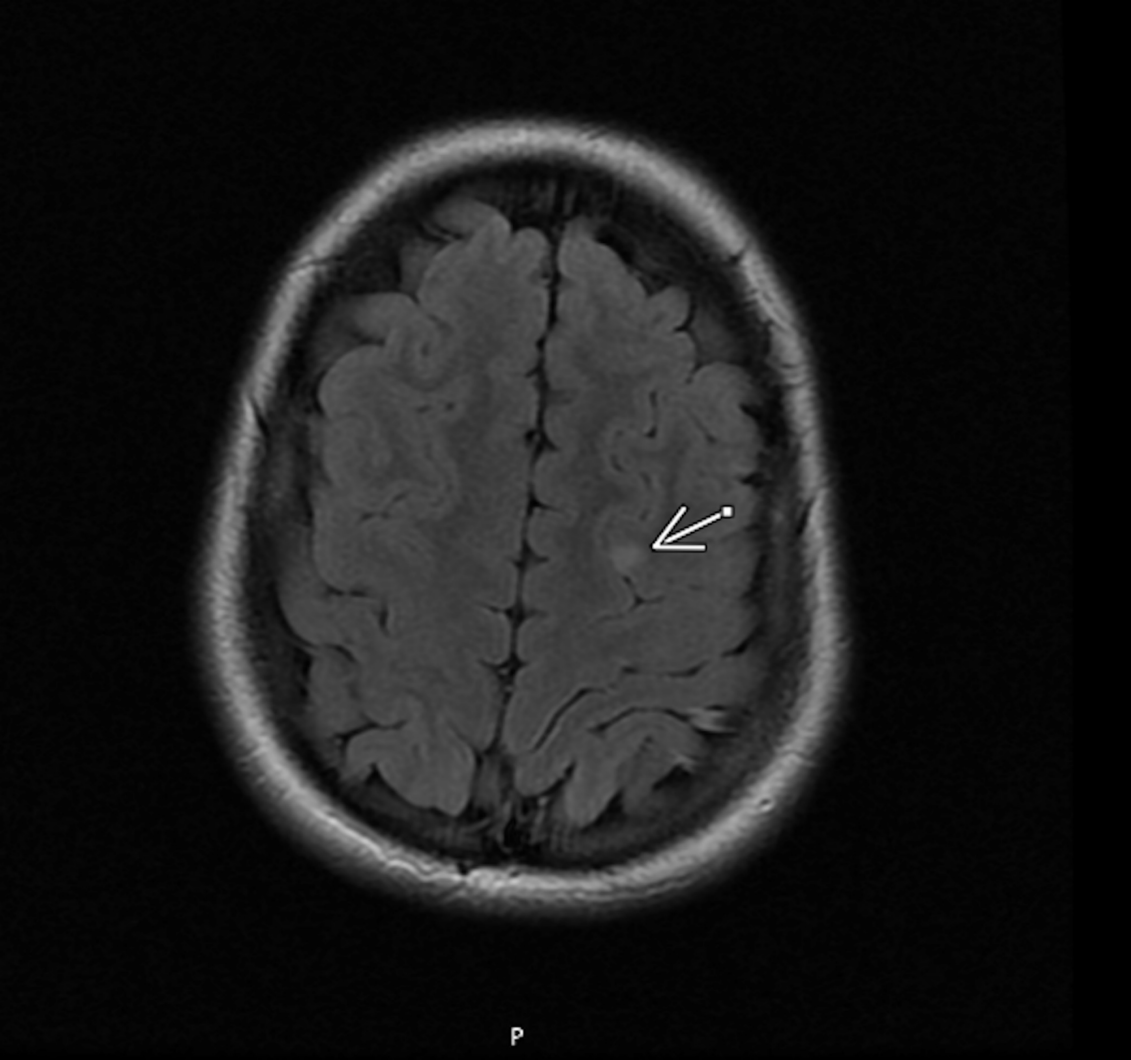
MRI brain without contrast: the arrow shows the lesion location Series 3 IMA 27, T2 flair

Her initial MRI with a contrast of the head was normal but in 18 months (Figure2); when her episodes intensified, a 5 x 5-mm-high left frontal lobe lesion was observed. This was read as frontal lobe cortical dysplasia versus slow-growing glioma, which continued to be stable with a three-month repeat MRI.

**Figure 3:**
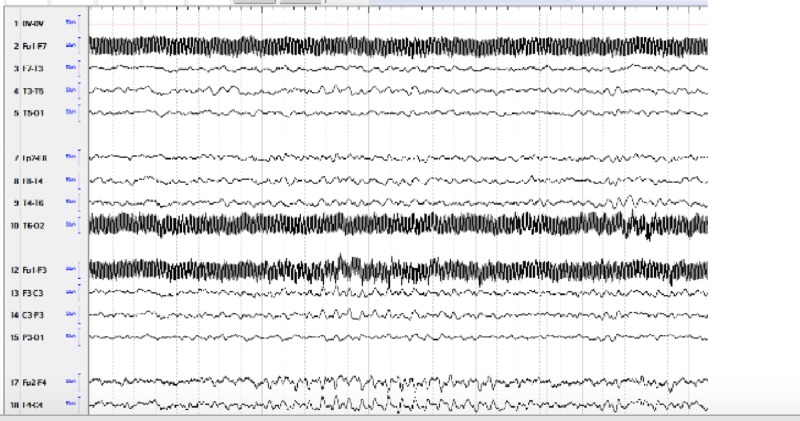
Continuous electroencephalogram Mild diffuse slowing, occasional brief right or bilateral frontal slowing, which may represent poorly formed spike-wave discharges, occasional to frequent right greater than left frontal (F4, F8) sharps

Although her initial 24-hour ambulatory EEG was read as normal, her video electroelectroencephalogram (vEEG) at a tertiary care epilepsy center (Figure 3) showed right more than left frontal (F4, F8) sharps and poorly formed generalized spike-wave discharges with a 10% to 20% chance of a seizure, but did not correlate with her observed behaviors leading to an inconclusive diagnoses but recommendations of continuing the 350 mg dose of topiramate. Although the incidents have decreased, she still has nocturnal enuresis. With topiramate, she was stable enough to move to a residential facility and continued to be stable and not requiring hospitalization for more than 18 months after her discharge from the last inpatient hospitalization. She continued to engage in therapy and was observed by a neurology service. The second opinion by another tertiary epilepsy service via medical records and parent interview only supported the diagnoses of NES. She has had subsequent serial MRIs over a period of 18 months and they showed a stable lesion.

## Discussion

Although several cases of diagnostic challenges with NES exist, this is the first study highlighting NES as multiple psychiatric hospitalizations, where monotherapy with topiramate prevented further hospitalizations. This case highlights the importance of timing of imaging with the worsening of clinical symptoms.

The question of whether the hospitalizations could be prevented early, given that she did present to an outpatient cardiology and neurology service, could be raised. It has been shown that prefrontal transcranial magnetic stimulation can cause bradycardia that could explain her vasovagal attacks [2]. Both seizures and NES are associated with greater autonomic activation in epileptic seizures than in NES, although more seizures in the NES group were associated with positive motor features [3]. She struggled with nocturnal enuresis that presented in the outpatient setting as urinary tract infections and also throughout her hospital stay, these episodes could be seen as nocturnal FLE [4].

Moreover, the MRI results showed foci that could explain it as a seizure, especially as the clinical symptoms and the remission with monotherapy of AED were consistent with how seizures may present. However, despite vEEG and consults from neurologists, no clinical correlation was observed with abnormal EEG. Nuances of vEEG have been discussed elaborately with its limitations highlighted with FLE, which may contribute to the inconclusiveness [5-7]. It may be argued that owing to the early onset and rapid spread of FLEs, studying the anatomoelectroclinical correlations of stereoelectroencephalography (SEEG) data may be the preferred the diagnostic modality [8]. However, her aggressive behavior did not make her an ideal candidate for the procedure. Given that FLE presents behaviorally and shows response to topiramate, valproic acid, and carbamazepine leading to remission, it is possible to consider it early in care. This may help with the possibility of step-down care from inpatients that may lead to an improvement in access to specialist care. Hence, the psychiatrist may consider this as a differential when presented with episodic events that have shown to have a pattern of increased intensity, with a shorter duration in between events. Moreover, topiramate has been shown to be effective in monotherapy in newly diagnosed FLE [9].

Limitations in reporting the case are highlighted in the episodic presentation. This case has been presented together herein; however, in the clinical setting, it was observed over time until a definite pattern was observed. Furthermore, although she was observed at three different hospitalizations, records from only two were obtained as the third center did not share the records. The second opinion obtained from the tertiary neurology team was based on the medical records rather than observation, as she was residing in different state.

## Conclusions

FLE has been poorly understood and often unrecognized by mental health professionals caring for children. Limited awareness of the behavioral manifestations may contribute to multiple inpatient hospitalizations. These can be a financial burden on both patients, family as well as the health care system. Appreciating the interplay of episodic behavioral symptoms specially in FLE may circumvent some of the burdens. Furthermore, this case highlights the limitations of the current diagnostic tools available in diagnosing frontal lobe seizures. She may be treated for the diagnosis of NES, and the frontal lobe lesion will always remain the primary focus and will require monitoring. Given the ambiguity, setting standards in the future for diagnoses among mental health professionals require a high index of suspicion for the psychiatric manifestations of FLE.
